# Identifying novel subgroups in heart failure patients with unsupervised machine learning: A scoping review

**DOI:** 10.3389/fcvm.2022.895836

**Published:** 2022-07-22

**Authors:** Jin Sun, Hua Guo, Wenjun Wang, Xiao Wang, Junyu Ding, Kunlun He, Xizhou Guan

**Affiliations:** ^1^Medical School of Chinese PLA, Beijing, China; ^2^Key Laboratory of Ministry of Industry and Information Technology of Biomedical Engineering and Translational Medicine, Chinese PLA General Hospital, Beijing, China; ^3^Medical Big Data Center, Chinese PLA General Hospital, Beijing, China; ^4^Department of Pulmonary and Critical Care Medicine, The Eighth Medical Center, Chinese PLA General Hospital, Beijing, China

**Keywords:** heart failure, subtype, machine learning, clustering analysis, scoping review

## Abstract

**Background:**

Heart failure is currently divided into three main forms, HFrEF, HFpEF, and HFmrEF, but its etiology is diverse and highly heterogeneous. Many studies reported a variety of novel subgroups in heart failure patients, with unsupervised machine learning methods. The aim of this scoping review is to provide insights into how these techniques can diagnose and manage HF faster and better, thus providing direction for future research and facilitating its routine use in clinical practice.

**Methods:**

The review was performed following PRISMA-SCR guideline. We searched the PubMed database for eligible publications. Studies were included if they defined new subgroups in HF patients using clustering analysis methods, and excluded if they are (1) Reviews, commentary, or editorials, (2) Studies not about defining new sub-types, or (3) Studies not using unsupervised algorithms. All study screening and data extraction were conducted independently by two investigators and narrative integration of data extracted from included studies was performed.

**Results:**

Of the 498 studies identified, 47 were included in the analysis. Most studies (61.7%) were published in 2020 and later. The largest number of studies (46.8%) coming from the United States, and most of the studies were authored and included in the same country. The most commonly used machine learning method was hierarchical cluster analysis (46.8%), the most commonly used cluster variable type was comorbidity (61.7%), and the least used cluster variable type was genomics (12.8%). Most of the studies used data sets of less than 500 patients (48.9%), and the sample size had negative correlation with the number of clustering variables. The majority of studies (85.1%) assessed the association between cluster grouping and at least one outcomes, with death and hospitalization being the most commonly used outcome measures.

**Conclusion:**

This scoping review provides an overview of recent studies proposing novel HF subgroups based on clustering analysis. Differences were found in study design, study population, clustering methods and variables, and outcomes of interests, and we provided insights into how these studies were conducted and identify the knowledge gaps to guide future research.

## Introduction

Heart failure (HF) is the serious manifestation and terminal stage of many cardiovascular diseases, with a high level of mortality and readmission rate (1). The global prevalence of HF is about 26 million, and with the aggravation of population aging and the increase of survival rate of acute coronary syndrome (ACS), the prevalence of HF is increasing continuously (2). However, the existing treatment measures are only symptomatic support treatments to improve symptoms, but cannot completely reverse the course of disease. One of the reasons for this phenomenon is that the current HF subpopulation cannot fully integrate the heterogeneity of HF clinical manifestations and progression, which further aggravates the serious consequences caused by inadequate or even inaccurate phenotypic classification.

In previous guidelines for heart failure, heart failure was classified according to the cut-off point of LVEF—heart failure with reduced ejection fraction (HFrEF): HF with LVEF ≤ 40%; Heart failure with preserved ejection fraction (HFpEF): HF with LVEF ≥ 50%; Heart failure with intermediate ejection fraction (HFmrEF): HF with LVEF > 40% and L VEF < 50% (3). The new guideline proposed a new and revised classification of HF according to LVEF: HF with improved ejection fraction (HFimpEF): symptomatic HF with a baseline LVEF ≤ 40%, a ≥ 10 points increase from baseline LVEF, and a second measurement of LVEF > 40%. When classifying heart failure based on LVEF, previous guidelines have used HFrEF and HFpEF, but for the types of heart failure with EF values between 40 and 49%, there are different terms used, and there is no uniform standard. In the new classification, patients with normalized EF may have decreased EF after drug treatment was discontinued, meaning that although EF improved, cardiac structure and function did not (4). Although large number of studies have analyzed and summarized the structural and functional characteristics of cardiac cells, intercellular excitation conduction pathway, and cellular inflammation degree of patients in each subtype under this classic classification, there is still a situation of lack of effective treatment and limited personalized medical care, which urgently requires more accurate and detailed grouping strategies (5, 6). The complexity of the development of heart failure is difficult to explain with the emphasis on symptoms and signs in the previous diagnostic classification. We believe that the new subtype will give new directions in the interpretation of heterogeneity and treatment selection. The introduction of subgroups of patients with homogeneous characteristics is helpful to treat patients according to their clinical and pathophysiological characteristics, reduces the complexity of the cross influence of data characteristics of different dimensions during the treatment of patients, and plays a role in improving the treatment and prognosis (7).

Machine learning (ML) has achieved good accuracy in early diagnosis, clinical classification and risk factor prediction of patients with HF (8, 9). However, because of the black box feature of the algorithm, we cannot learn from the classification process of the algorithm. Unsupervised machine learning, specifically clustering analysis, is used to find the similar or different features between patients groups, and identify subgroups with homogeneous features. Clustering studies have certain advantages in characterizing, classifying or treating patients differently. Clustering algorithms commonly are performed in a static way with baseline data and/or outcome data. They are useful to answer descriptive questions (10, 11). In the early attempts, unsupervised clustering analysis algorithms were used on clinical laboratory indexes and demographic data characteristics of patients with heart failure to make homogeneous inductive groups (12, 13). In recent studies, researchers also used echocardiography, genomics and comorbidity characteristics to explore more grouping strategies (14). Without knowing the outcomes information (i.e., unsupervised learning), clustering analysis can comprehensively reflect the association between new subgroups and heart failure outcomes and other prognostic indicators.

There are wide variations in studies defining new heart failure subgroups, in study design, statistical methods, and reporting of outcomes, which makes comparing and summarizing results from different studies very difficult. Therefore, it is necessary to conduct a scope review to summarize the current practice in studies on the new subgroups of heart failure, clarify the limitations and provide direction and planning for the future research. At present, some researchers have discussed the application of machine learning in heart failure subtypes. Banerjee et al. included 15 studies published up to 2015, and compared the symptoms of cardiovascular diseases such as ACS, myocardial infarction (MI) and heart failure (HF) (15); In addition, Banerjee and others evaluated the subtype definition and risk prediction of ML in HF, ACS and AF (Atrial Fibrillation), and systematically reviewed them (15). However, in their research, the definitions of heart failure and subgroups are only a part of the research, and the clustering variables concerned are not comprehensive, and the included research is up to December, 2019 at the latest. Therefore, it is necessary to define the scope with a specific focus on the subtype classification in heart failure, and fully incorporating the latest research reports. This scoping review will integrate the current evidence on subtype classification of heart failure reported in the existing literature to provide a reference for clinicians and community health care workers to manage HF better, and identify the knowledge gaps to point out the direction for future research.

## Methods

This scoping review followed the Preferred Reporting Items for Systematic reviews and Meta-Analyses extension for Scoping Reviews (PRISMA-ScR) guideline (16), and a competed PRISMA-ScR checklist was provided in Supplementary material. A study protocol was designed by a senior author and agreed by all authors, and this protocol was not registered or published.

### Literature search

We performed a search in PubMed to identify primary studies on discovery of new HF sub-types by using clustering analysis. The search strategy contained 3 modules: the HF module, the algorithm module, and the sub-type module, and a filter of publication time till 31st December 2021 (see Supplementary material Search Strategy).

### Eligibility criteria

Studies were included if they defined new subgroups in HF patients using clustering analysis methods. Exclusion criteria were: (1) Reviews, commentary, or editorials, (2) Studies not about defining new sub-types, (3) Studies not using unsupervised algorithms.

### Study selection

Titles and abstracts were independently scanned by one of the two authors and checked by the other, to identify potentially eligible articles, which were then assessed with full texts for final inclusion. Disagreements were resolved through discussion by the two reviewers, and a third author made the final decision when an agreement was not reached.

### Data extraction

Data was collected on basic study characteristics including title, name of the first author, year of publication, country, and information and the analysis and results including study population, sample size, clustering method(s), types of clustering variables, and outcome(s). The data extraction form and data extracted in this study can be found in the Supplementary material. All included articles were reviewed and extracted by one of the two authors and double checked by the other. Disagreements were resolved through discussion, if necessary the final judgment was from a third reviewer.

### Data synthesis

Data synthesis was performed with descriptive statistics and data visualization. Categorical variables were presented as counts and proportions, and continuous variables were presented with median and IQR. All the statistical analyses were performed with R version 3.6.1 and RStudio version 1.2.5001, and packages ggplot2, networkD3 (sankey diagram), ggparliament (parliament diagram), UpSetR (upset plot) and scatterpie (bubble chart).

## Results

### Search finding

**FIGURE 1:**
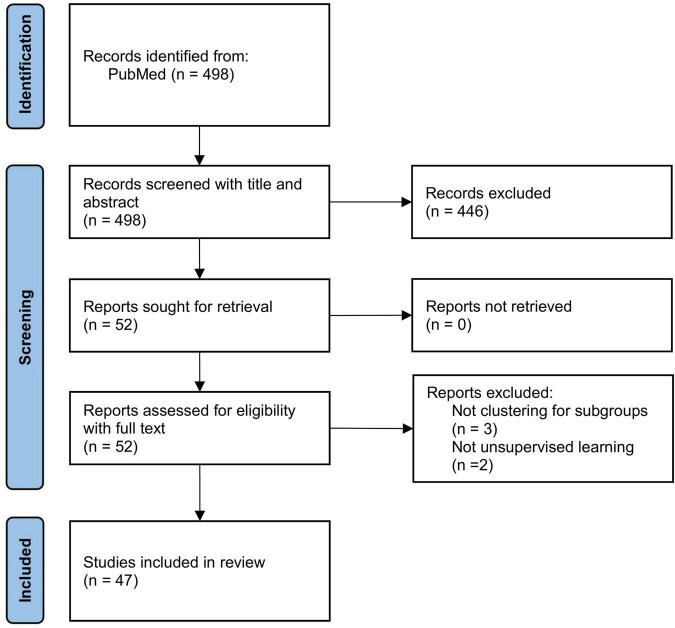
PRISMA flow diagram for study inclusion.

A total of 498 studies were identified by the search strategy, all of them were screened for titles and abstracts, of which 446 were excluded at this stage. Fifty-two studies entered the stage of full-text reading to assess their qualification, and five of them were excluded, for reasons shown in Figure 1. In the end, 47 studies were included in the review.

### Characteristics of the included studies

**FIGURE 2:**
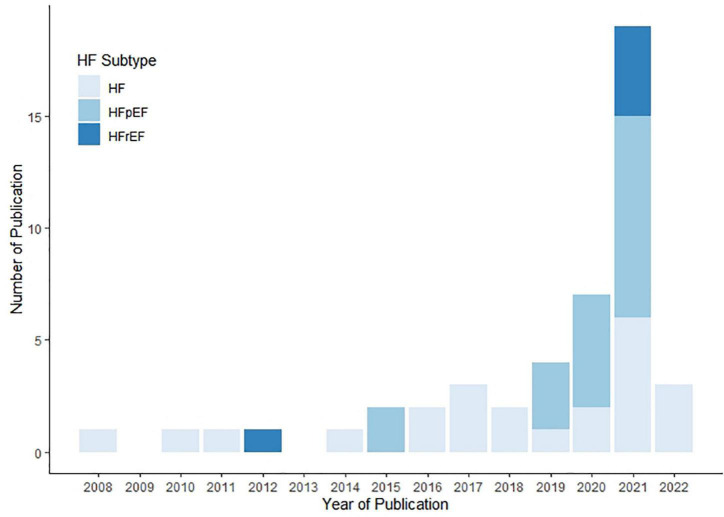
Number of publication per year by HF sub-types.

Table 1 shows the basic characteristics of the included studies in this review. Among the 47 included studies, 23(48.9%) studies focused on patients with generalized HF (7, 13, 17–31), while the rest 24 studies focused on patients with specific categories of HF, among which 19 studies (40.4%) focused on patients with HFpEF (32–50), and the other 5 studies (14, 51–54) (10.6%) focused on HFrEF. Of these studies, only one (2.1%) was published before 2010 (55) and four (8.5%) were published between 2010 and 2014 (19, 20, 54, 56). Most of the research was published after 2015, and the research after 2020 accounted for 61.7% (32, 34, 35, 37, 38, 40, 42–44), as shown in Figure 2.

**TABLE 1:** Descriptive statistics of study characteristics.

Characteristics	Studies, *n* (%)/M(Q1–Q3)
***HF subtype***	
HF	23 (48.9%)
HFpEF	19 (40.4%)
HFrEF	5 (10.6%)
***Years published***	
<2010	1 (2.1%)
2010–2014	4 (8.5%)
2015–2019	13 (27.7%)
≥2020	29 (61.7%)
***Author country***	
Asia	5 (10.6%)
Europe	18 (38.3%)
America	23 (48.9%)
Oceania	1 (2.1%)
***Data source***	
Asia	4 (8.5%)
Europe	14 (29.8%)
America	21 (44.7%)
Oceania	1 (2.1%)
Multinational	7 (14.9%)
***Data types***	
EHR	24 (51.1%)
RCT	9 (19.1%)
Disease registries	8 (17.0%)
Observational data	3 (6.4%)
Claims data	1 (2.1%)
EHR and claims data	1 (2.1%)
EHR, RCT, and registries data	1 (2.1%)
***Method***	
K-Means/Medoids	9 (19.1%)
LCA	11 (23.4%)
Hierarchical	22 (46.8%)
Hierarchical & K-Means/Medoids	1 (2.1%)
SOM	1 (2.1%)
Spectral	1 (2.1%)
Mixture model-based	2 (4.3%)
*Sample size*	480 (301–1619)
*Number of variables*	18 (11–47)
*Number of clusters*	3 (3–6)
***Variable type***	
Demographic	24 (51.1%)
Clinical	25 (53.2%)
Laboratory	21 (44.7%)
Imaging	24 (51.1%)
Genetic	6 (12.8%)
Symptoms and complaints	18 (38.3%)
Comorbidities	29 (61.7%)
***Outcome***	
Cross sectional	7 (14.9%)
Mortality	36 (76.6%)
Hospitalisation	27 (57.4%)
Other events	14 (29.8%)
***External validation***	
Yes	6 (12.8%)
No	41 (87.2%)

**FIGURE 3:**
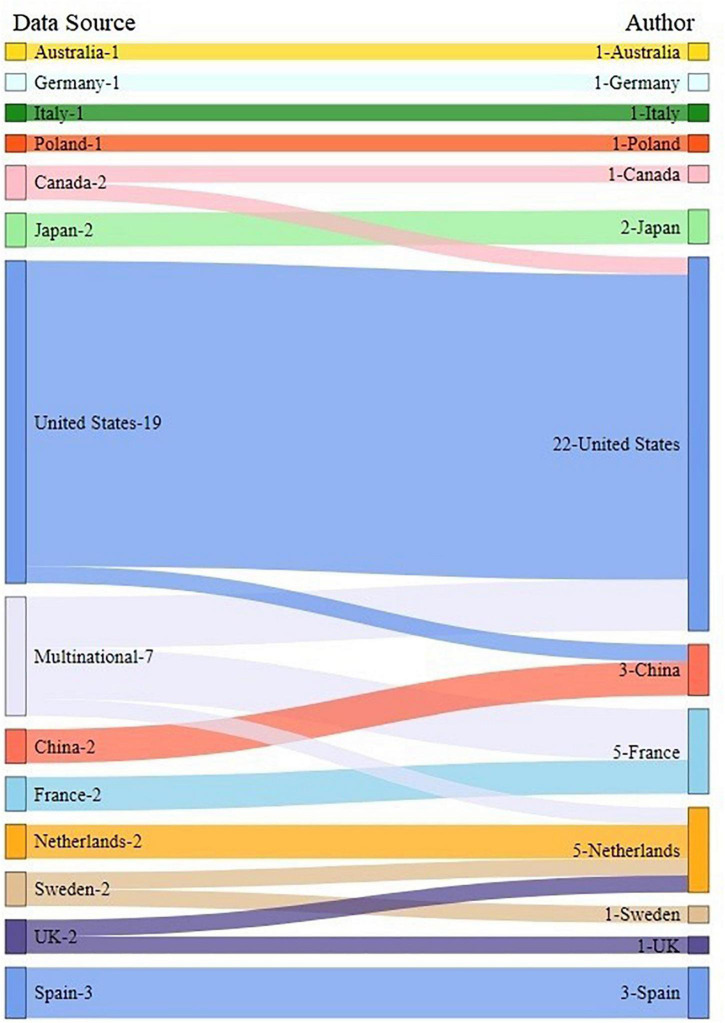
Relationship between data sources and corresponding authors.

In all the included studies, the corresponding authors were from 13 different countries, including the United States (22, 46.8%) (18–22, 24, 26–28, 33, 36, 37, 40, 41, 44, 48, 53, 54, 56–59), the Netherlands (5, 10.6%) (23, 45, 47, 55, 60), France (5, 10.6%) (13, 17, 32, 43, 51), Spain (3, 6.4%) (25, 34, 38), China (3, 6.4%) (7, 35, 49) and Japan (2, 4.3%) (31). Australia (52), Germany (30), Italy (14), Poland (39), Switzerland (46), Canada (50) and the United Kingdom (29) each had only one study (1, 2.1%). The research data were from a single country in 40 studies (85.1%), and the rest 7 studies (14.9%) were performed with multinational data (13, 17, 24, 28, 37, 43, 60). The relationship between data sources and corresponding authors is shown in Figure 3. We further classify them according to their continents, and find that the highest number of authors and participants are from America and Europe, followed by Asia and Oceania, as shown in Table 1.

We analyzed the types of data source, the results showed that in all included in the article, there are 24 articles (51.1%) using EHR data, 9 articles (19.1%) using RCT research data, 8 articles (17.0%) using disease registration data, 3 article (6.4%) using the observational data, 1 article (2.1%) using the claims data. In addition, one study used EHR data and Claims data simultaneously, and another study used EHR data, RCT data and registries data simultaneously.

In addition, of the 47 included studies, only 6 (12.8%) were externally validated, while the remaining 41 studies (87.2%) were not.

### Types of clustering methods in the included studies

**FIGURE 4:**
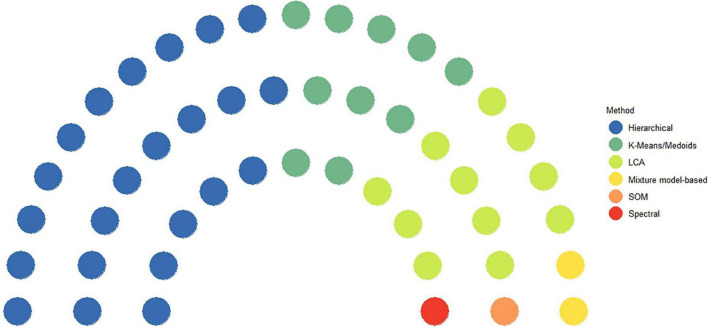
Types of machine learning methods used in identifying HF subgroups.

The clustering methods were categorized into six main types, and the usage of each type of methods in the included studies are shown in Figure 4 and Table 1. The most commonly used ML method was hierarchical clustering method (32–33, 35, 38–41, 43, 44), accounting for 46.8% (22/47) of all studies, followed by latent class analysis (11, 23.4%) (7, 17, 24, 26, 29, 30, 32, 36, 37, 47, 55) and K-Means/Medoids (9, 19.1%) (13, 28, 31, 34, 42, 50, 52, 53, 60), and two studies used mixture model-based approach (4.3%) (46, 58). The least commonly used methods were spectral (49), self-organizing map (23) and composite of hierarchical and K-Means/Medoids (27).

### Types of clustering variables used in the included studies

**FIGURE 5:**
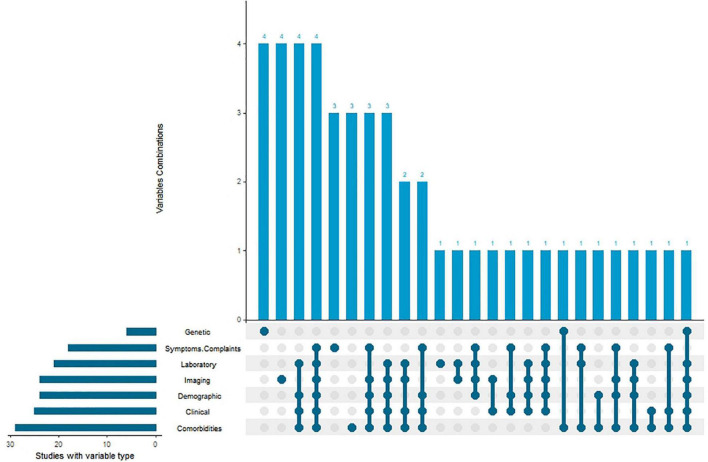
Types of clustering variables used in identifying HF subgroups.

We divide all the variables used by the institute for unsupervised cluster analysis into seven categories: demographic data (such as gender, age, education level, etc.), clinical data (such as heart rate, respiratory rate, etc.), laboratory data, image features (such as LEVF, etc.), genetic data, clinical symptoms and complications, and comorbidities. We have sorted out the frequency with which these variable types are used and whether they are jointly used in cluster analysis, as shown in Table 1 and Figure 5. As far as the frequency of variable types is concerned, comorbidities are the most frequently used in these studies 29(61.7%), and the clinical data, imaging data, demographic data and laboratory data are almost the same, which are 25(53.2%), 24(51.1%), 24(51.1%) and 21(44.7%) respectively. Among them, gene data, symptoms and complications data, image data, laboratory data and complications data are used alone in the process of sub-grouping in some studies. Most studies combine multiple data types to make a new subgroup classification of heart failure.

### Sample size, number of clustering variables, and number of clusters

**FIGURE 6:**
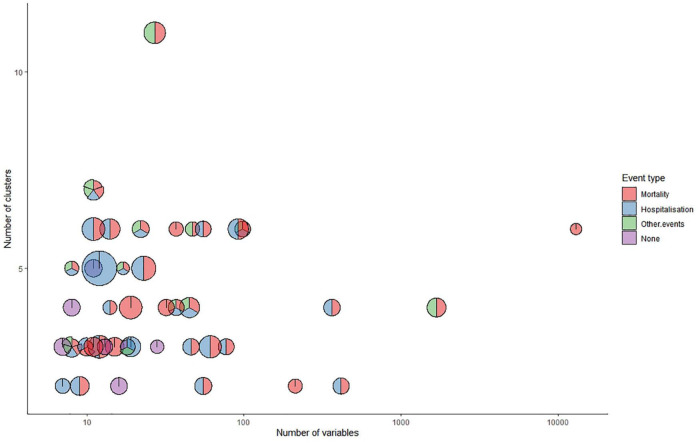
Features of the clusters identified in the included studies.

The sample sizes in the derivation of subgroups ranged from 63 to 318,384. In all 47 studies, the median sample size was 480, of which 23 studies (48.9%) included data sets of more than 500 people, and only 2 studies (4.3%) had a sample size of less than 100 people. The number of clustering variables involved in the research also varies widely, the research with the least variables was using only 7 clinical, laboratory or imaging indicators, and the research with the most variables was using 13,000 genes for clustering analysis to determine their molecular subgroups. The number of clusters obtained in most studies ranged from 2 to 7 (97.9%), with a median of 3, of which 16 studies (34.0%) finally got 3 clusters, and only one study got 11 clusters. Figure 6 shows the relationship between the number of variables (X-axis, in log scale) and the number of clusters (Y-axis) identified in each study, and the sample size (in log scale) is presented as the radius of the bubble. The Spearman correlation was −0.14 between sample size and number of clustering variables, 0.15 between sample size and number of clusters, and 0.13 between number of clustering variables and number of clusters.

### Prognostic implications of the clusters proposed in the included studies

Many different outcomes were used to evaluate the prognostic implication of the identified new subgroups, thus they were classified into four categories: death, hospitalization, other events, and cross-sectional study (i.e., no prognostication was assessed). The most commonly used endpoints were death (36, 76.6%) and hospital (27, 57.4%) respectively, while in 7 studies (14.9%) no analysis was performed for prognostic implications. The outcomes evaluated in each study are shown in Figure 6.

## Discussion

In this scoping review, we summarized the current research on identifying subgroups in HF patients with unsupervised machine learning methods. This type of studies increased quickly over past years, and there were 19 new publications in 2021. Differences were found in study design, study population, clustering methods and variables, and outcomes of interests, and we aimed to provide insights into how these studies were conducted and identify the knowledge gaps to guide future research.

Most of the studies were conducted by researchers from developed countries, or in geographic, from Europe and North America, which is not a surprising finding given their leading position in the field of biomedical and clinical researches. However, subgroups identified from these populations may have poor generalizability in other part of the world. Africa, South America, South and West Asia were under represented, since the data availability is limited in those areas. We also noticed that most researchers worked on data from their own country, and only a few studies were from multinational collaboration. Future research should consider combining datasets containing patients from different countries and regions, to investigate the potential application of the new subgroups worldwide.

With regarding to the clustering methods, in this scoping review, the most commonly used unsupervised machine learning algorithm is hierarchical clustering, followed by K-Means or K-Medoids. Hierarchical clustering has the advantage of not requesting predefined number of clusters, which is useful in exploring novel subgroups. Researchers have had long history in using K-Means or K-Medoids clustering in analyzing data, and these methods were considered as multivariate analysis before the term of machine learning getting its popularity (61, 62).

The novel HF subgroups can be defined with different types of variable, however, the application of subgroups also depends on how difficult these clustering variables can be collected. Obtaining demographic variables and underlying comorbidities is straightforward by asking the patient’s medical history at admission, which made them as the most frequently used variables in clustering analysis, and laboratory data and imaging data can also be obtained in routine examination after admission. However, genomics or proteomics data may need extra special examination methods, which are less common compared with other data types, so that genomics or proteomics are rarely used in the included studies, but this also heralds the great potential of genomics in revealing the prognosis of heart failure patients.

The implementation of machine learning methods relay on data in a large degree. In the included studies, 23 studies used datasets of less than 500 patients and only 15 studies used data sets of more than 1,000 patients. At the same time, the number of clustering variables is relatively big, sometime even higher than the sample size, which may lead to overfitting issues. A negative correlation was observed between the number of clustering variables and the sample size, which is not as expected. When more clustering variables are included in the analyses, researchers need to make sure the sample size is sufficient to get reliable results.

Some included studies did not evaluate the prognostic implication of the proposed subgroups, and we marked these studies as cross-sectional studies. Unsupervised cluster analysis does have obvious advantages in finding out the heterogeneity among patients, and the new subgroup is also more accurate in describing the symptoms and complications of patients, but not connecting with the prognosis means that it is limited in clinical application, so we hope that more researches will make a clear plan for the clinical endpoint of patients. In addition, we found that few studies have set the quality of life or daily behavior ability as the research endpoint, which may be due to the fact that similar endpoints need more detailed evaluation scales or multi-dimensional evaluation indicators, which are quite different in the nature of easy access compared with the outcome endpoints such as death or readmission.

Unlike traditional prediction models, which pay more attention to the prediction accuracy and absolute probability of having a specific event, clustering analysis focused on classifying complex and heterogeneous diseases and identifying people with similar clinical characteristics. Thus, subgroups identified with clustering analysis may have better explanation and clinical meaning than prediction models. With these novel subgroups, patients can be more accurately risk stratified by more simple and easily available clinical indicators, and then targeted treatment schemes can be formulated.

With the development of coronary intervention technology, more patients with coronary heart disease survive and develop into heart failure. Coupled with the aggravation of population aging, the number of patients with heart failure is increasing year by year (63, 64), there is a higher proportion of elderly patients among them, and the existence mode of comorbidity is more complicated, which is followed by the increase of medical expenses, mortality and hospitalization rate (65). More and more researchers are aware of the importance of comorbidity management. The study on comorbidity of patients with heart failure found that the number of participants suffering from diabetes, chronic kidney disease and atrial fibrillation was higher (66), and other common comorbidities included hypertension and chronic obstructive pulmonary diseases. Some studies showed that some comorbidities would change the disease phenotype of patients with heart failure, and even become the main cause of heart failure (67), whereas some studies reported that the comorbidity of patients with heart failure was more serious, which might be caused by heart failure. Diabetes, hypertension, etc., are also associated with worse clinical outcomes in other diseases. Therefore, it is of great significance to carry out more personalized management for patients with heart failure under different comorbidity modes. These common comorbidities are important variables in our included studies, and the emergence of new subgroups and new treatment standards have also brought about the improvement of clinical prognosis in these studies. In addition to the elderly patients with heart failure, recent studies have found that the prevalence of cardiovascular comorbidities in middle-aged patients with heart failure is also very high, compared with the elderly patients with heart failure (>85 years old) (68). In the included studies, the new grouping of patients based on comorbidity or combined with other types of data also provided reference for clinical treatment.

There are also some limitations of the current scoping review. First, when searching for eligible publications, we only performed the literature search in PubMed database. Some other databases such as scienceDirect, Embase, IEEE, Scopus, etc., were not searched specifically, since most of the relevant publications are covered by PubMed, and looking for more databases will only increase the duplicates and add unnecessary workload. Given this is a scope review rather than a systematic review, we strictly enforce this search strategy, and we are confident the results presented in this scoping review are not biased. Second, we only included publications in English, and excluded those in other languages, which may reduce the diversity of this scope review. Third, we did not evaluate the evaluation criteria and external validation of the novel subgroups, since they are seldom done in the included studies. We believe validation or replication of the proposed subgroups are essential before these subgroups will be used in clinical practice, and future studies should pay more attention to these analyses. At last, this scoping review is only a comprehensive description of the existing researches on subgroup identification in HF patients, thus no formal assessment on methodological quality (or risk of bias) or meta-analysis was performed in this review. These analyses are usually within a systematic review, and are beyond the scope of this scoping review. In future research, we plan to perform a systematic review on studies with similar subgroup definition and a meta-analysis on their prognostic performance.

## Data availability statement

The original contributions presented in this study are included in the article/Supplementary material, further inquiries can be directed to the corresponding authors.

## Author contributions

JS and HG screened the publications, extracted data, and prepared the first draft. WW conducted a rigorous review of the first and final drafts. XW and JD participated in the revision of the research protocol and data extraction form. KH and XG designed and conceptualized the research project, rigorously revised and approved the final manuscript. All authors contributed to the article and approved the submitted version.

## Conflict of interest

The authors declare that the research was conducted in the absence of any commercial or financial relationships that could be construed as a potential conflict of interest.

## Publisher’s note

All claims expressed in this article are solely those of the authors and do not necessarily represent those of their affiliated organizations, or those of the publisher, the editors and the reviewers. Any product that may be evaluated in this article, or claim that may be made by its manufacturer, is not guaranteed or endorsed by the publisher.
