# Supplementary material for: Identifying novel subgroups in heart failure patients with unsupervised machine learning: A scoping review
Source: Front Cardiovasc Med. 2022 Jul 22;9:895836. doi: 10.3389/fcvm.2022.895836 (PMC9353556; doi:10.3389/fcvm.2022.895836)
Supplement: Supplementary file 1 [file Table_1.DOCX]

Supplementary Material

1 Search strategy HF

(("heart failure"[MeSH Terms] OR "heart failure"[Title/Abstract]) AND

("cluster analysis"[MeSH Terms] OR "machine learning"[MeSH Terms] OR "unsupervised"[Title/Abstract] OR "clustering"[Title/Abstract] OR "latent class"[Title/Abstract]) AND

("subgroup*"[Title/Abstract] OR "subpopulation*"[Title/Abstract] OR "subtyp*"[Title/Abstract] OR "cluster*"[Title/Abstract] OR "phenotyp*"[Title/Abstract])) AND

(1000/1/1:2021/12/31[pdat])

2 Supplementary table: Data dictionary of supplementary data file

| Column | Variable Name | Description |
| --- | --- | --- |
| A | ID | Manuscript ID of publications included for full text check |
| B | Title | Title of publication |
| C | Author | First author of publication |
| D | Year | Year of publication |
| E | HF Subtype | Heart failure subtype: HF; HFrEF; HFpEF |
| F | Author Country | Country of corresponding author |
| G | Data Country | Country of research data |
| H | Method | Name of clustering method |
| I | Method_short | Name of clustering method in short |
| J | Sample Size Training | Sample size of training dataset |
| K | Total Number of Variables | Total number of variables used in clustering |
| L | Demographic | Whether Demographic variables were used: 1, Yes; 0, No |
| M | Clinical | Whether Clinical variables were used: 1, Yes; 0, No |
| N | Laboratory | Whether Laboratory variables were used: 1, Yes; 0, No |
| O | Imaging | Whether Imaging variables were used: 1, Yes; 0, No |
| P | Genetic | Whether Genetic variables were used: 1, Yes; 0, No |
| Q | Symptoms.Complaints | Whether Symptoms or Complaints variables were used: 1, Yes; 0, No |
| R | Comorbidities | Whether Comorbidities variables were used: 1, Yes; 0, No |
| S | Number of Clusters | Number of clusters identified |
| T | cross sectional | Only cross sectional analysis (no outcome): 1, Yes; 0, No |
| U | Mortality | Outcome of mortality was evaluated: 1, Yes; 0, No |
| V | Hospitalisation | Outcome of hospitalisation was evaluated: 1, Yes; 0, No |
| W | Other events | Other outcomes were evaluated: 1, Yes; 0, No |
